# *Adenomatous polyposis coli* mutants dominantly activate Hsf1-dependent cell stress pathways through inhibition of microtubule dynamics

**DOI:** 10.18632/oncotarget.4513

**Published:** 2015-07-22

**Authors:** Alexander E. Davies, Kaitlyn Kortright, Kenneth B. Kaplan

**Affiliations:** ^1^ Department of Cell and Molecular Biology, University of California, Davis, CA, USA

**Keywords:** cell stress, adenomatous polyposis coli, microtubules, cancer field effect, Hsp90

## Abstract

Cancer cells up-regulate cell stress pathways, including the protein chaperone Hsp90. Increases in Hsp90 are believed “buffer” mutant protein activities necessary for cancer phenotypes. Activation of the cell stress pathway also alters the transcriptional landscape of cells in ways that are critical for cancer progression. However, it is unclear when and how the cell stress pathway is de-regulated during cancer progression. Here we report that mutations in *adenomatous polyposis coli* (APC) found in colorectal cancer activate cell stress pathways in mouse intestinal crypt cells, prior to loss of heterozygosity at APC or to the appearance of canonical intestinal cancer markers. Hsp90 levels are elevated in normal APC heterozygote crypt cells and further elevated in non-cancer cells adjacent to dysplasias, suggesting that the Hsp90 stress pathway marks the “cancer-field” effect. Expression of mutant APC in normal human epithelial cells is sufficient to activate a cell stress pathway via perturbations in microtubule dynamics. Inhibition of microtubule dynamics is sufficient to activate an Hsf1-dependent increase in gene transcription and protein levels. We suggest that the early activation of this Hsf1 dependent cell stress pathway by mono-allelic mutations in APC can affect cell programming in a way that contributes to cancer onset.

## INTRODUCTION

The heat shock response is part of larger network of pathways that responds to cell stress and that plays an essential, conserved role in cellular adaptation following perturbations to cell homeostasis. A variety of studies indicate that cancer cells depend on a similar adaptive response [1, 2]. Indeed, cells lacking HSF1, the master transcriptional regulator of the heat shock pathway, are refractory to transformation by variety of oncogenes [1, 3]. These findings suggest a role for the heat shock pathway in cancer initiation or progression. Although the exact mechanisms by which the heat shock pathway aids in oncogenic transformation remain unclear, one possibility is that increases in heat shock proteins (HSPs), such as Hsp90, contribute to the proteomic stability. Hsp90 has been proposed to act as a “buffer” to maintain the activity of mutated oncogenes. Support for this idea comes from the ability of Hps90 to interact with and stabilize a wide-range of oncogenic proteins (e.g., v-Src and Raf) [2, 4–8]. Evidence suggests that Hsp90 and associated co-chaperones also play a more general role in coping with proteomic stress that arises from amplified genes, increases in chromosome ploidy, or deregulation of transcriptional regulators [9–12]. The need to maintain multiple protein networks involved in signaling and cell survival may further contribute to the proteomic stress experienced by cancer cells. Thus, cancer cells may have a large number of Hsp90 targets, an idea supported by the complexity of the Hsp90-interactome [13–16], and by the finding that a larger percentage of Hsp90 is in an active conformation in cancer cells [17–19].

Although Hsp90 is the best studied downstream target of the heat shock response, its levels and the expression of a large number of stress respsonsive genes are under control of the master transcriptional regulator, HSF1. Recent work has revealed that HSF1, and by extension its network of regulated genes, plays a critical role in cancer onset and progression. This is strikingly demonstrated in studies that have deleted HSF1 from mice carrying cancer-promoting mutations [6, 20–23]. Loss of HSF1 in p53−/− mice reduces the incidence of lymphomas and animals instead succumb to sarcomas and carcinomas [1, 19]. In contrast, Ras activation induces HSF1 and loss of HSF1 delays tumor formation [1, 3, 24–27]. The distinct roles of HSF1 in specific cancers suggest that its targets vary depending on the genetic context of the cell. Support for this idea comes from the finding that HSF1 interacts with distinct sets of promoters in the context of heat shock response as compared to the conditions in a malignant cancer cell [4, 6, 28]. The implication is that the heat shock pathway and HSF1 responds differentially to evolving cellular conditions and that chronic activation of this pathway may allow cells to adapt to a cancer state. We will refer to the activation of this HSF1 dependent transcriptional response as part of a larger cellular stress response, or pathway.

The necessity of the HSF1 cell stress pathway in carcinogenesis is clear. However, the upstream triggers that activate it and when activation is required during carcinogenesis are not well understood [9, 29]. One hypothesis is that cell stress pathway activation is triggered in response to a single, initiating mutation in a cancer pathway. This possibility is consistent with cell stress and cell senescence induced by oncogenic mutations and the ability of HSF1 to repress senescence [13, 15, 16, 30, 31]. This hypothesis predicts that cancer driver mutations will result in chronic activation of the HSF1 cell stress pathway prior to cancer onset to allow cells to tolerate additional genomic instability and thus contribute to cancer progression. As a first test of these predictions, we focused on driver mutations in the *adenomatous polyposis coli* (APC) tumor suppressor that contribute to both familial and sporadic human colorectal cancer [17, 19, 32]. APC mutants most frequently give rise to truncated proteins that we have previously shown to dominantly inhibit microtubule dynamics, resulting in chromosome segregation errors [19, 20, 22, 28]. Mitotic errors were also observed in the otherwise normal intestinal crypt cells of APC^Min/+^ mice, arguing that microtubule dynamics are perturbed due to a single mutant allele of APC [19, 33]. APC^Min/+^ mice develop adenomas (or dysplasias) in the small intestine, which exhibit loss of heterozygosity at APC and an elevation in β-catenin levels, consistent with proposed progression of human colorectal tumors [24–27, 34]. We tested the possibility that a monoallelic mutation at APC is sufficient to activate the heat shock pathway, possibly by inhibiting microtubule dynamics. We found that Hsp90 levels are elevated both in abnormally organized crypts (i.e, dysplasias) that have undergone LOH as well as in histologically normal tissue adjacent to the dysplasia. Remarkably, the majority of normal intestinal crypt cells from APC^Min/+^ mice have elevated Hsp90 compared to wild type intestinal cells. Significantly, we found that *in vitro* expression of APC mutants, or direct perturbations of microtubule dynamics, activate a cell stress response, arguing for a direct link between APC mutants, microtubule dynamics and the cell stress program. The findings argue that a single cancer-associated mutation can activate a cell stress pathway in a way mimics the phenotype of mature cancer cells in otherwise normal cells, thus anticipating the cancer cell state.

## RESULTS

### Hsp90 is elevated in APC^Min/+^ crypts prior to cancer onset

**Figure 1:**
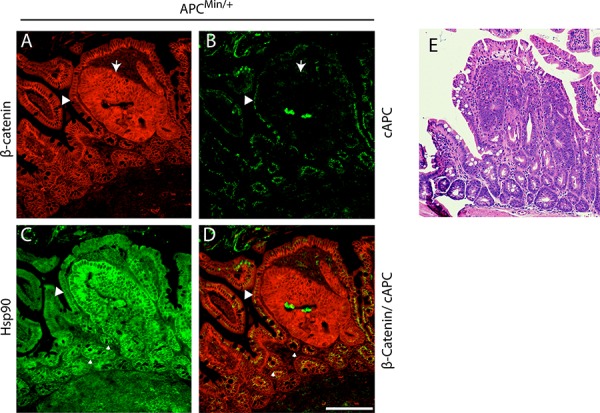
Hsp90 is elevated in APC^Min/+^ early cancer lesions in the small intestine APC^Min/+^ small intestinal tissue section displaying a dysplasia and surrounding histologically normal tissue. **A.** β-Catenin (red) staining outlines the basolateral membrane of cells and increased levels of cytoplasmic levels delineate dysplastic regions of the intestinal crypt (see arrow). **B.** A carboxy terminal antibody to APC (cAPC) only recognizes full length APC (i.e., not the APC^Min^ protein; green signal) and thus identifies cells that have lost expression of the full length APC protein, an event that corresponds with increases in β-Catenin (arrow). **C.** Serial sections were stained to show Hsp90 levels (green). **D.** An overlay of β-Catenin and cAPC staining is shown in red and green, respectively. Small arrows denote regions of ‘high’ Hsp90 adjacent to the adenoma that express wild type APC. Scale bar = 20 um. **E.** H&E stained serial section of the intestinal region presented in (A) through (D) at 4x magnification.

**Figure 2:**
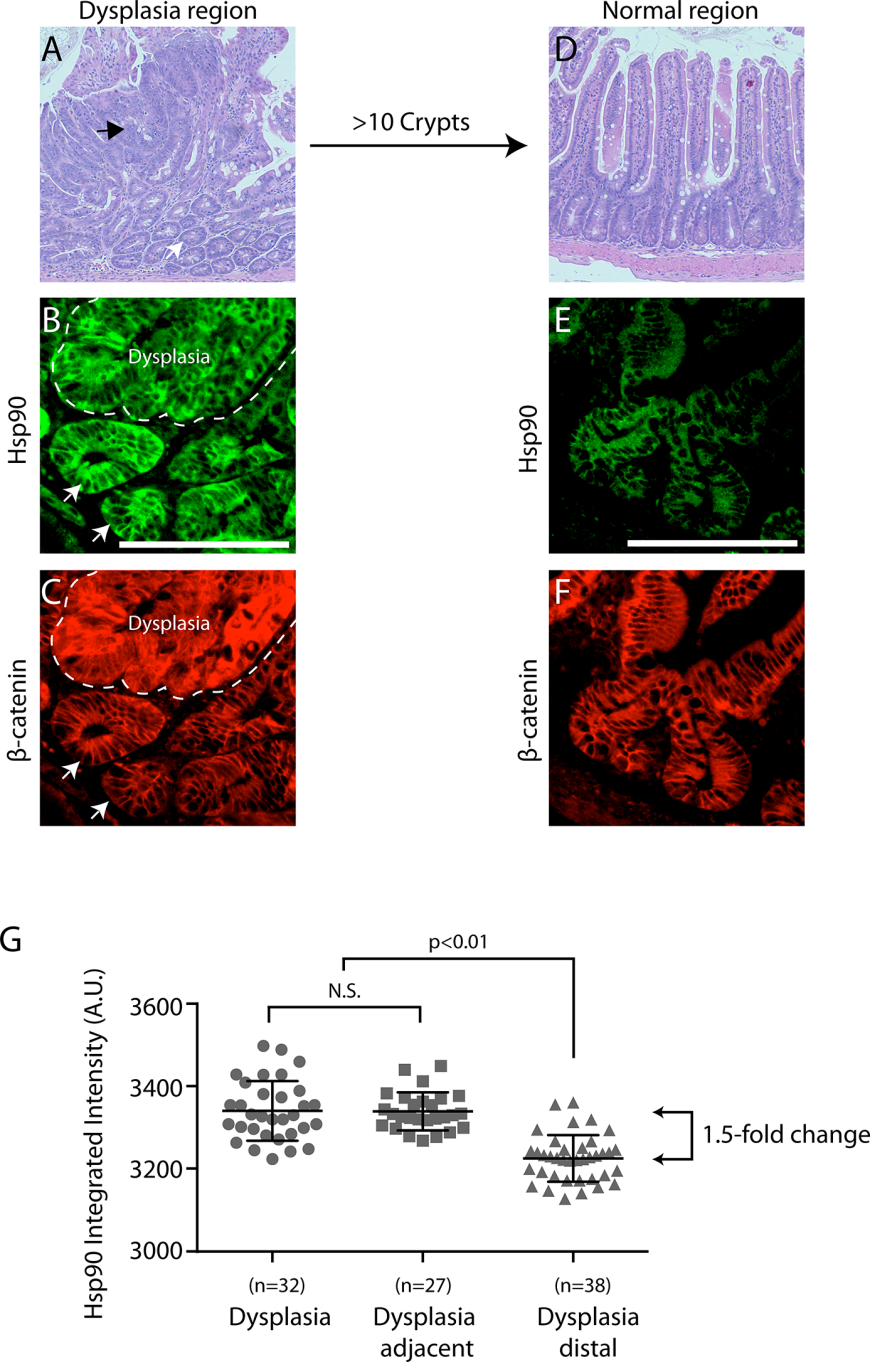
Hsp90 is elevated in normal cells adjacent to early cancer lesions - a “field effect” **A.** Representative APC^Min/+^ small intestinal tissue section highlighting regions of dysplasia (i.e., altered cell organization and expanded cell number) by H&E staining at 4x magnification (see black arrow indicates dysplasia, white arrow indicates adjacent tissue). A different dysplastic region was co-stained for β-catenin (**B.** red) and Hsp90 (**C.** green; dysplasia delineated by dashed line). Arrows indicate cells with normal levels of β-Catenin and high levels of Hsp90. **D.** A normal region of the intestine approximately 10 crypts away from any dysplasia was visualized with H&E staining at 4x magnification. A different normal region was stained for β-catenin (D, red) and Hsp90 (**E.** green). All images are exposed and adjusted identically. Scale bar = 20 uM. **F.** Scatter plot of fold change in Hsp90 levels relative to normal APC^Min/+^ crypts as indicated. Each point represents the Hsp90 intensity signal from a single dysplasia or crypt as described in Materials and Methods. Error bars represent the standard deviation from the mean. The *p*-value shown for comparison indicated by brackets; N.S. indicates no statistical significance. The arrowed brackets indicate fold-change between the indicated mean intensity values.

To assess the temporal relationship between cell stress pathway activation and cancer onset, we analyzed the levels of Hsp90 in three regions in APC^Min/+^ mice that are representative of discreet disease states: (i) dysplastic regions are associated with early cancer and characterized by cell expansion, disorganized columnar epithelium, increases in β-catenin and loss of full length APC (Figure 1A–1B, arrow); (ii) regions adjacent to dysplasias, usually within 1–20 cell equivalents that appear otherwise normal (e.g., normal levels of β-catenin; Figure 1A–1B, arrowhead); (iii) normal intestinal crypts found approximately 10 crypt distances away from any dysplasia. Consistent with expectations, dysplastic regions exhibited elevated levels of β-catenin as compared to normal adjacent tissue (Figure 1A) and the absence of full length APC, as detected with a carboxy terminal antibody that does not recognized the Min mutant protein [28, 35, 36] (Figure 1B, see large arrowhead). In adjacent serial sections, we found that Hsp90 levels are dramatically elevated within dysplastic tissues (1.5-fold increase; Figure 1C), as compared to histologically normal intestinal crypts from the same animal. Unexpectedly, we found that Hsp90 levels are equally elevated in a subset of normal cells adjacent to dysplastic tissues (Figure 1C, see small arrowheads). These were judged to be non-cancer cells based on the low levels of β-catenin and normal apical localization of full length APC (Figure 1D, see small arrowheads). Additionally, histological analysis showed no evidence of altered cell morphology or invasion into the normal region from the dysplasia, nor was there evidence of inflammatory infiltrate (Figure 1E). In total, 60% of the dysplasias analyzed (*n* = 25) (Figure 2A, representative H&E image) showed regions of elevated Hsp90 in dysplasia adjacent cells with low levels of β-catenin (Figure 2B (see arrows), Figure 2C). In contrast, cells in regions of normal intestine distal from dysplasias (> 10 crypts away, Figure 2D) exhibited uniform and lower levels of Hsp90 and β-catenin (Figure 2E and 2F). Quantification of Hsp90 fluorescence intensities in dysplasias compared to normal cells adjacent to dysplasias showed no significant difference in levels, yet these levels were 1.5-fold greater than in normal cells greater than 10 crypts away (Figure 2G). Consistent with previous reports, we could not detect Hsp70 signal above background in normal cells in the small intestine and there was no detectable increase in staining in dysplastic tissue ([22, 29]; data not shown, see Discussion). We conclude that Hsp90 is up-regulated prior to malignant tumor formation in pre-cancerous dysplastic regions as well as in neighboring, non-cancer cells. Although we cannot rule out that the change in normal cells is caused by secreted signals from the nearby dysplasia, the fact that not all normal cells exhibit elevated Hsp90 argues for an intrinsic cell change.

**Figure 3:**
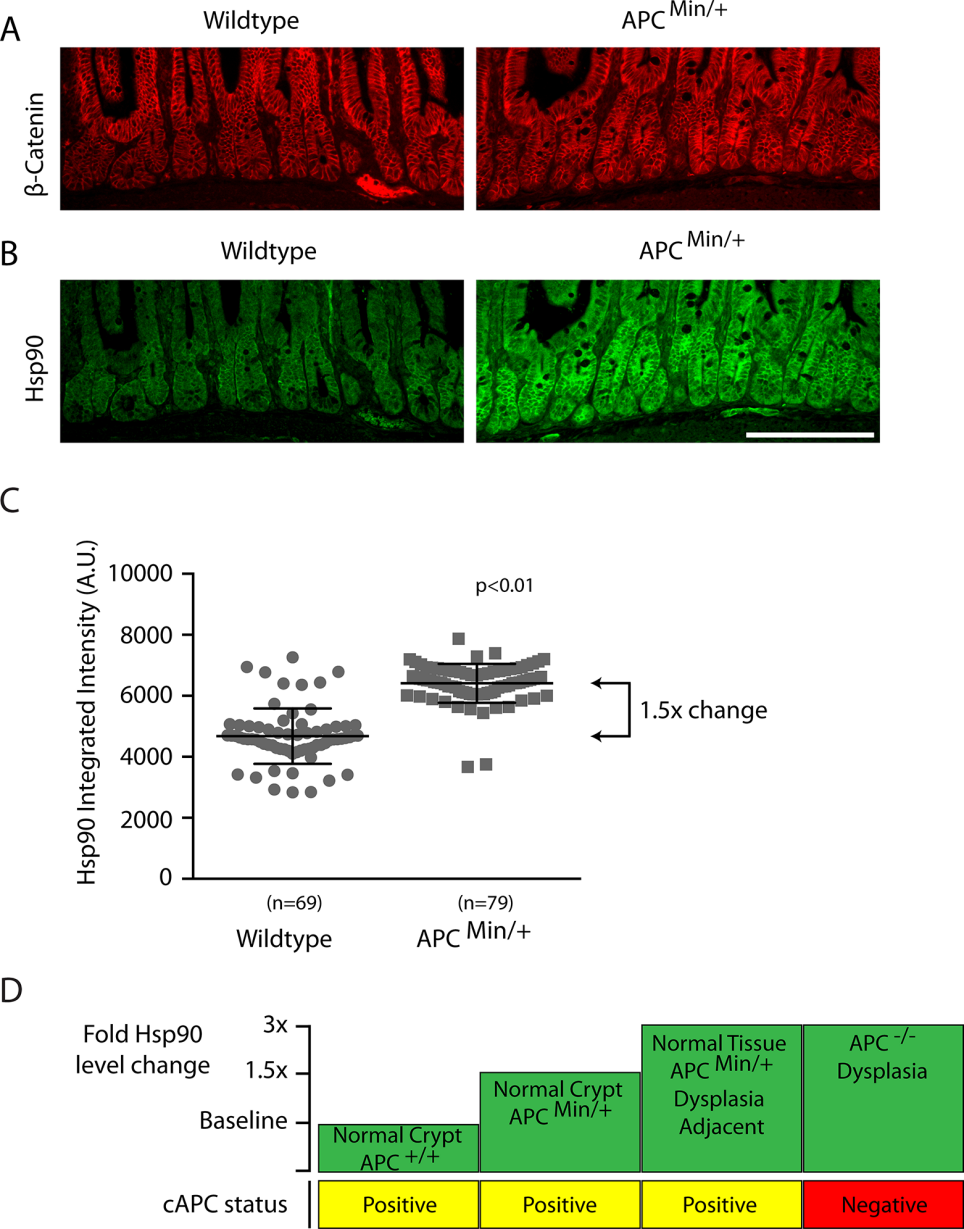
Hsp90 levels are globally elevated in normal intestinal cells from APC^Min/+^ compared to wild type APC^+/+^ mice Low magnification views of representative small intestinal crypt sections from wild type or APC ^Min/+^ mice as indicated and stained for **A.** β-catenin, or **B.** Hsp90. Hsp90 levels where exposed and adjusted identically. Scale bar = 20uM. **C.** Scatter plot of Hsp90 total integrated fluorescence intensity from single crypts (*n* = 69, 79) expressed as arbitrary fluorescent units (A.U.). Measurements were taken within the transit amplifying compartment using a fixed sized region of interest in ImageJ software (see materials and methods). Error bars represent the standard deviation; the *p*-value was obtained from a Student's *T*-test. **D.** A diagram depicts the relative fold-changes in Hsp90 (green boxes) between normal and pre-cancerous lesions in the small intestines of APC^+/+^ and APC^Min/+^ animals compared to the status of full length APC expression (yellow/red boxes).

One possibility is that the APC^Min^ mutation is directly linked to the elevated levels of Hsp90 found in both normal and dysplastic intestinal cells. To test this idea, we compared levels of Hsp90 in normal crypts in wild type (i.e., APC^+/+^) and APC^Min/+^ intestines. To ensure that we analyzed non-dysplastic crypts in APC^Min/+^ intestines, we selected histologically normal regions with low levels of β-catenin that express full length APC (Figure 3A and Supplementary Figure 1). We found elevated levels of Hsp90 in all histologically normal crypt and villus cells in APC^Min/+^ compared to wild type intestines (Figure 3B, 3C), albeit at lower than levels found in dysplastic tissue. To summarize, Hsp90 levels are found in three distinct tiers in mouse small intestinal cells (Figure 3D). Compared to the baseline levels of Hsp90 found in APC^+/+^ intestines, there is a 1.5-fold increase in Hsp90 in otherwise normal APC^Min/+^ crypt cells prior to loss of heterozygosity at APC and another 1.5-fold increase in dysplastic cells or in cells adjacent to the dysplasia (a total of a 3-fold increase; Figure 3D). The magnitude of Hps90 increase is significant and comparable to *in vivo* studies of a variety of cancers and other physiological stresses [6, 30, 31]. The association of elevated Hsp90 with the APC^Min^ allele prior to cancer onset, supports the idea that APC mutations are directly responsible for activating cell stress pathways prior to cancer onset.

### APC mutants that inhibit microtubule dynamics activate a cell stress response

**Figure 4:**
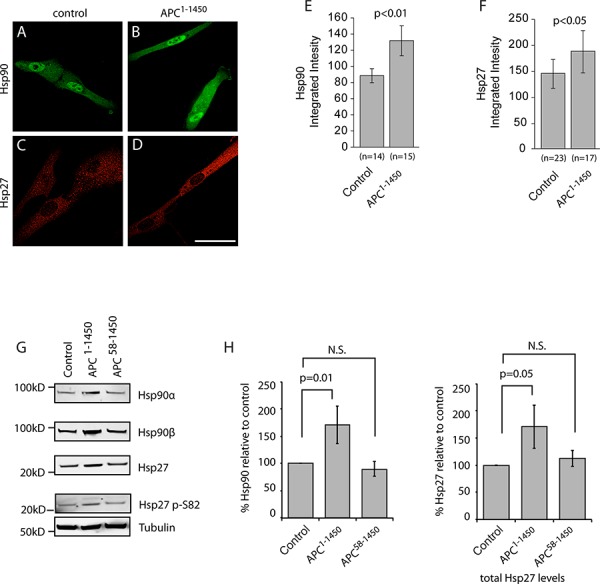
APC^1–1450^ dominantly induces heat shock proteins in normal human epithelial cells hTERT-RPE cells were transiently transfected with **A.** and **C.** CMV-myc (control) or **B.** and **D.** CMV-myc-APC^1–1450^ (APC^1–1450^) and stained with (A and B) anti-Hsp90 antibodies or (C and D) anti-Hsp27 antibodies (red). Scale bar = 20 uM. **E.** Total integrated intensities were determined for (E) Hsp90 and **F.** Hsp27 in a fixed region of interest using ImageJ software. All values are expressed as arbitrary fluorescence intensity units (A.U.) and error bars represent the standard deviation values. **G.** Western blots using the indicated antibody were performed on the soluble protein fraction from cell lysates of hTERT-RPE cells that had been transiently transfected as indicated. The nearest relevant molecular weight marker is indicated to the left of the panels. **H.** Western blot analyses of Hsp90 and Hsp27 from the indicated cell extracts were analyzed from multiple, independent experiments. Fluorescent signals were quantified and values are reported with *p* values for the indicated, bracketed comparisons.

Min mice, as in the majority of human colorectal cancers, express a monoallelic APC mutation that encodes for a truncated protein and this single allele is sufficient to drive the formation of intestinal adenomas [32, 37]. We have previously shown that truncating APC mutations found in human colorectal cancers (e.g., APC^1–1450^; expressing the first 1450 amino acids) dominantly inhibit microtubule dynamics by forming a hetero-oligmoer with full length APC that inhibits the activity of the microtubule plus-end binding protein EB1 [19, 22, 23, 28]. We confirmed the effect of APC^1–1450^ on microtubules in a normal diploid epithelial cell line (hTERT-RPE) by expressing APC^1–1450^, thus mimicking the APC heterozygous state. We found that microtubules were less organized in hTERT-RPE cells expressing APC^1–1450^ and that EB1 was observed more consistently along the length of microtubules, as opposed to strictly in comets at microtubule plus ends. This change is consistent with there being fewer growing microtubules (see Supplementary Figure 2A–2B) and thus altered microtubule dynamics [33, 38]. To test if the changes in microtubule dynamics induced byAPC^1–1450^ also activates the cell stress pathway, we measured Hsp90 levels by immunofluorescence using pan-Hsp90 antibodies. Measurements 72 hours post-transfection showed that expression of APC^1–1450^ significantly increases Hsp90 levels in the cytosol and the nucleus compared to control transfection (∼48% increase; compare Figure 4A, 4B and 4E). Similarly, we also found that levels of the cytosolic chaperone, Hsp27, are increased after expression of APC^1–1450^ compared to control transfection (∼29% increase; Figure 4C, 4D and 4F). To verify the changes in protein levels observed by fluorescence microscopy, we performed immunoblots. We found both Hsp90 alpha, Hsp90 beta, and Hsp27 were elevated in cells expressing APC^1–1450^ compared to control transfections (Figure 4G). The increase in Hsp levels was reproducible and are consistent with increases observed after acute heat stress in mammalian cells and with exposure of hTERT-RPE cells to heat stress (∼1.5–2.0-fold increase, Figure 4H; unpublished results and [34, 39]). In contrast, Hsp70 and β-catenin levels remain unaffected under these conditions (Supplementary Figure 3A and 3C). Significantly, we also observed an increase in Hsp27 phosphorylation at S82, an activating modification that has been shown to be sensitive to changes in microtubule dynamics (Figure 4G, [35, 36, 40–42]).

To test whether APC^1–1450^ activates the cell stress pathway via inhibition of microtubule dynamics, we used an allele with a second mutation in the oligomerization domain, APC^58–1450^. APC^58–1450^ fails to interact with full length APC and thus does not inhibit microtubule dynamics [22, 43, 44]. We reasoned that if the increase in HSP levels induced by APC^1–1450^ is mediated through a dominant alteration in microtubule dynamics, transfection of APC^58–1450^ should no longer increase HSP expression. Consistent with this prediction, expression of APC^58–1450^ failed to increase levels of Hsp90 alpha, Hsp90 beta, and Hsp27, as well as Hsp27 S82 phosphoyrlation (Figure 4G). Based on these results we conclude that APC^1–1450^ is sufficient to dominantly activate an arm of the cell stress pathway that may be specific to cancer (see Discussion), possibly by inhibiting microtubule dynamics through the EB1 pathway.

### Inhibition of microtubule dynamics is sufficient to activate the cell stress response

**Figure 5:**
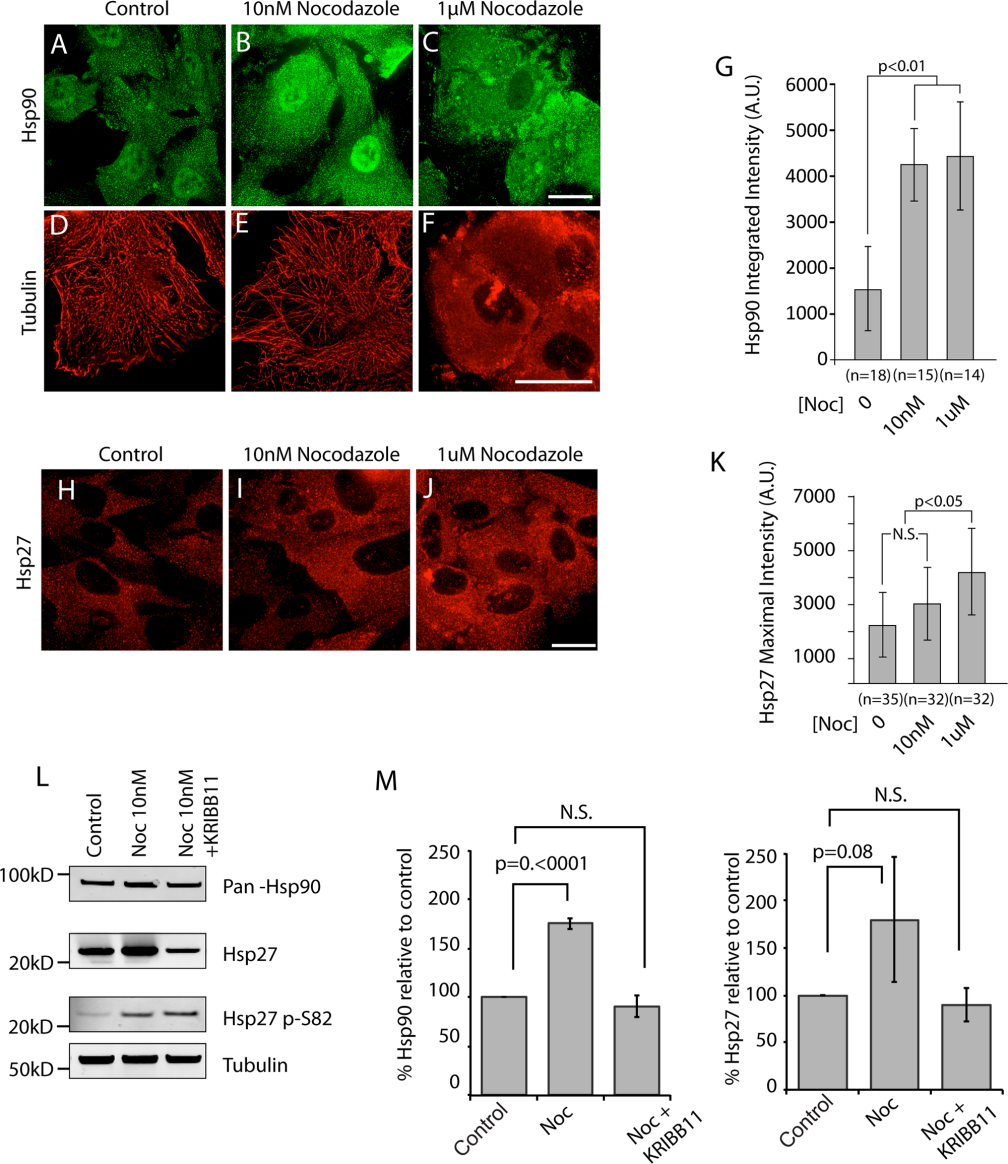
Inhibition of microtubule dynamics is sufficient to activate cell stress pathway in hTERT-RPE cells **A–F.** hTERT-RPE cells were treated with the indicated amounts of the microtubule destabilizing drug, nocodazole to inhibit dynamics (B and E, 10 nM) or to depolymerized the microtubule network (C and F, 1 μM) and representative cells are shown co-stained for Hsp90 (A–C) and for α-tubulin (D–F). **H–I.** In a separate experiment, Hsp27 was also detected. The total integrated fluorescent intensities for **G.** Hsp90 and **K.** Hsp27 were measured in the cytoplasm using ImageJ software as described in Materials and Methods. Values are expressed in fluorescence intensity units (A.U.) and error bars represent the standard deviation values. N.S. indicates no statistical significance. **L.** Western blots were performed on the soluble fraction of cell lysates from hTERT-RPE and after the indicated treatments. The nearest relevant molecular weight marker is indicated to the left of the panels. **M.** Western blot analyses of Hsp90 and Hsp27 from cells treated as indicated were analyzed from multiple, independent experiments. Fluorescent signals were quantified and values are reported with *p* values for the indicated, bracketed comparisons. Scale bar = 20 uM.

It is possible that this arm of the cell stress pathway responds to subtle changes in filament dynamics or to gross perturbations in the organization of the microtubule network, or to both. Consistent with a role for filament dynamics and with our previous work, we found that siRNA of EB1, the microtubule plus-end binding protein inhibited by APC^1–1450^ is sufficient to activate the cell stress pathway. Both Hsp90 and Hsp27 levels are increased relative to control and Hsp27 p38-kinase modification increases (see Supplementary Figure 2C–2E, p-S82). To further address the role of microtubule dynamics in cell stress pathway activation, we used either micromolar or nanomolar concentrations of the microtubule poison, nocodazole to differentially perturb microtubule function. In hTERT-RPE cells treated with DMSO as a control, Hsp90 distributes evenly between the cytosol and nucleus and microtubules form a robust interphase array (Figure 5A and 5D). As expected, treatment of hTERT-RPE cells with 10nM nocodazole for 12 hours to inhibit filament dynamics left the microtubule network intact (Figure 5E). Significantly, these conditions gave rise to a consistently observed 3-fold increase in the levels of cytoplasmic and nuclear Hsp90 that was confirmed by immunoblot (Figure 5B, 5G, 5L and 5M). We also observed an increase in the levels of Hsp27 in the cytosol that was confirmed by immunoblot, although the magnitude of the increase varied between experiments (Figure 5H–5L and 5M). The increase in the levels of phosphorylated Hsp27 is similar to that observed after expression of APC^1–1450^ and consistent the idea that changes in microtubule dynamics activate a p38-stress kinase pathway (Figure 5L). Addition of 1uM nocodazole resulted in a complete depolymerization of the microtubule array (Figure 5F) but did not further increase the levels of Hsp90 (Figure 5C, 5G and 5L). In contrast, Hsp27 levels further increase when the microtubule network is completely disrupted (i.e, 1 uM nocodazole, Figure 5J and 5K). Together, these findings argue that this cell stress pathway responds to subtle perturbations in microtubule dynamics. Finally, we confirmed that Hsp70 and β-catenin levels were not affected under any of these conditions; we therefore conclude that APC mutants trigger a stress response, distinct from a classic heat shock response, largely through the inhibition of microtubule dynamics (Supplementary figure 3B and 3C).

The range and magnitude of changes in Hsp90 and Hsp27 as well as the specificity of the response raise the possibility that decreases in microtubule dynamics leads to an HSF1-dependent change in gene transcription, akin to that observed in cancer cells [6, 28]. We tested this possibility using the HSF1 inhibitor, KRIBB11. KRIBB11 interferes with the ability of HSF1 to recruit transcriptional elongation factors and prevents stress induced increase in HSP gene transcription [37, 45–48]. Co-incubation of cells with 10nM nocodazole with KRIBB11 completely blocked the increase in Hsp90 and Hsp27 compared to control cells (Figure 5L and 5M). Consistent with the specific action of KRIBB11, Hsp27 p-S82 levels were not affected, indicating that phosphorylation is upstream of HSF1 activity. Together, these results support a pathway whereby APC mutants alter microtubule dynamics that in turn increase HSF1 activity and activate a global cell stress response.

### APC mutants and inhibition of microtubule dynamics induce Hsf1 activation

**Figure 6:**
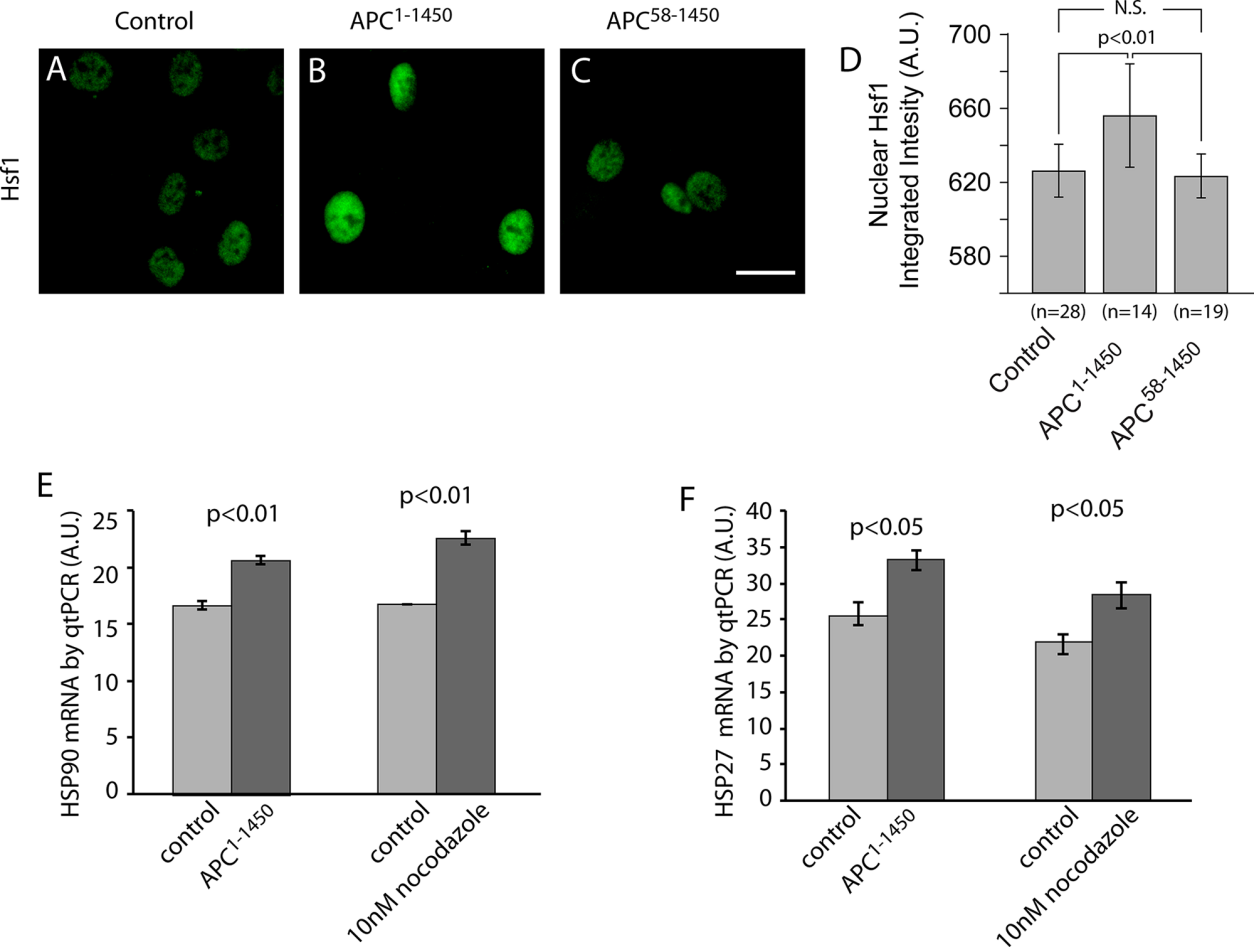
Inhibition of microtubule dynamics activates heat shock factor 1 (Hsf1) and heat shock protein gene transcription **A–C.** hTERT-RPE cells were stained with Hsf1 antibodies after transient transfection with (A) CMV-myc (Control), (B) CMV-myc-APC^1–1450^ (APC^1–1450^), or (C) CMV-myc-APC^58–1450^ (APC^58–1450^), as indicated. **D.** Total integrated fluorescent intensities were measured for nuclear Hsf1 using ImageJ software as described in the Material and Methods. Scale bar = 20 uM. The indicated treatments were performed on hTERT-RPE and then **E.** Hsp90 and **F.** Hsp27 mRNA levels were measured using RT-qPCR. Levels of GAPDH mRNA were used to standardize the total amount of mRNA between samples and levels represent relative levels of the indicated mRNA.

Hsf1 regulation is complex but is thought to involve the stress induced de-repression of the Hsf1 monomer state leading to the formation of Hsf1 trimers, followed by nuclear enrichment and a series of less well-understood post-translational modifications [23, 49]. To assess the activation state of Hsf1 under conditions where microtubule dynamics are inhibited, we used immunofluorescence to characterize its nuclear localization. We first tested hTERT-RPE cells expressing APC^1–1450^. In control transfections, we observed low levels of nuclear Hsf1 (Figure 6A). However, in cells expressing APC^1–1450^ we observed a significant increase in the levels of Hsf1 in the nucleus (Figure 6B and 6D). Importantly, in cells expressing APC^58–1450^, the double mutant that fails to inhibit microtubule dynamics, nuclear levels of Hsf1 did not increase, a result that is consistent with the failure of this mutant to elevate levels of Hsp90 and Hsp27. Treatment of cells with siRNA against EB1 or low dose nocodazole (10 nM) to inhibit microtubule dynamics both resulted in elevated levels of nuclear Hsf1 (Supplementary Figure 4A and 4B).

As a direct test of Hsf1 activity on target genes, we analyzed mRNA levels of Hsp90 and Hsp27 using quantitative PCR in hTERT-RPE cells. We observed a significant increase in Hsp90 and Hsp27 transcripts in cells expressing APC^1–1450^ or treated with low dose (10 nM) nocodazole (Figure 6E and 6F), consistent with the magnitude of change previously reported during heat shock pathway activation [38, 50, 51]. In contrast, siRNA of EB1 resulted in detectable, but not statistically significant, increases in HSP transcripts (Supplementary Figure 4C). This finding is consistent with the less robust increase in HSP protein levels observed after siRNA against EB1. We conclude that inhibition of microtubule dynamics through low dose nocodazole treatment, or expression of APC^1–1450^, is sufficient to increase transcription and expression of Hsp90 and Hsp27 through the activation and nuclear localization of Hsf1.

**Figure 7:**
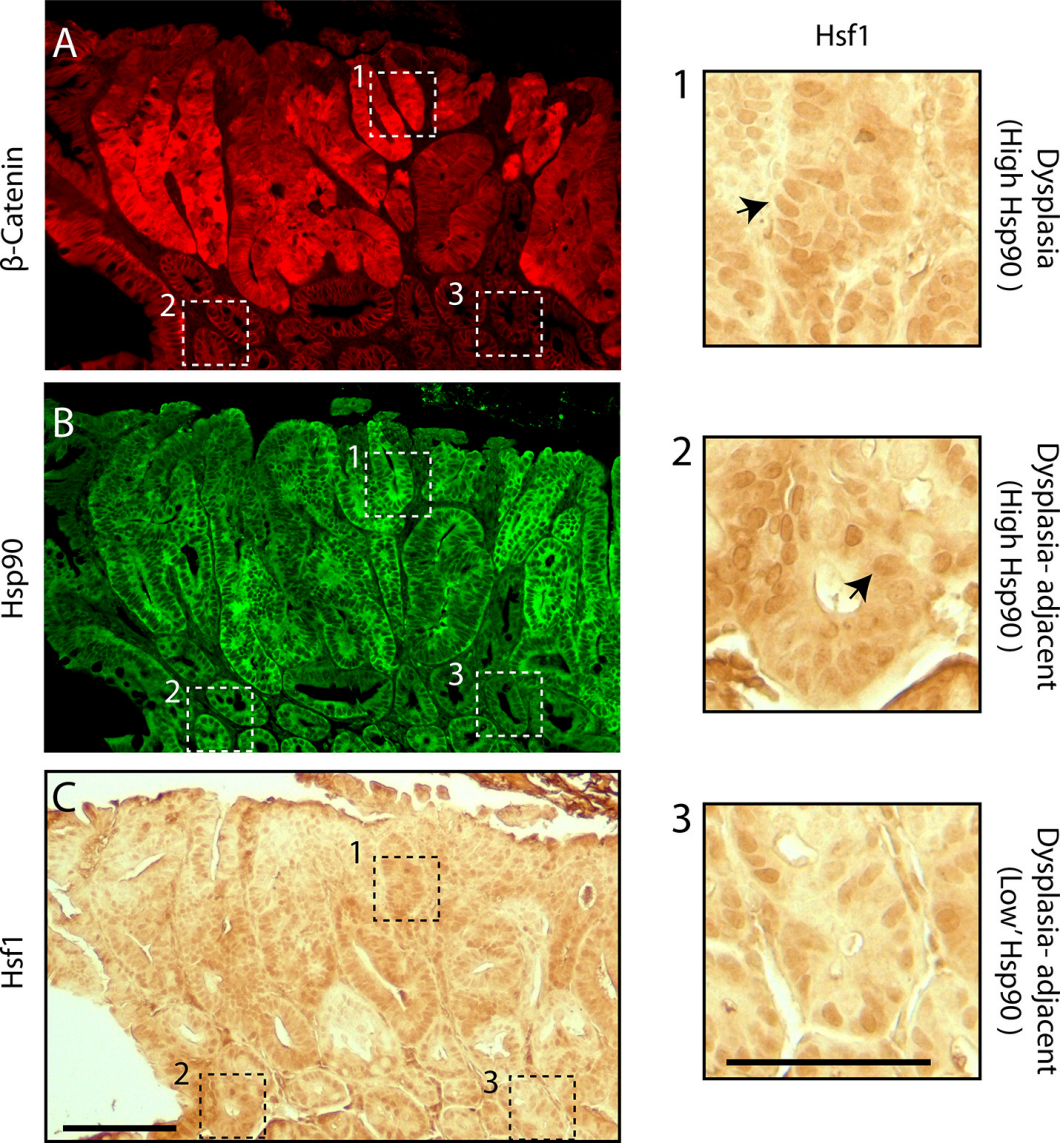
Hsf1 nuclear enrichment correlates with levels of Hsp90 in normal and pre-cancerous cells in APC^Min/+^ intestinal crypts Dysplastic regions were identified in APC^Min/+^ small intestines using **A.** β-catenin and these regions along with surrounding histologically normal tissue were also stained for **B.** Hsp90 (green). **C.** An adjacent section of the same region was stained using immunohistochemistry with antibodies against Hsf1. Scale bar = 20 uM. Dashed boxes outline distinct regions of the tissue. Box1 shows a region with high levels of β-catenin and boxes 2 and 3 are regions with lower levels of β-catenin. Equivalent regions shown by the dashed boxes are presented as 4-fold magnified images. Arrows indicate nuclear staining of Hsf1. Scale bar = 10 uM.

The relationship between Hsf1 and cancer initiation led us to examine the behavior of Hsf1 in intestinal epithelial cells in APC^Min/+^ animals. Unfortunately, we were unable to reliably observe Hsf1 using fluorescence microscopy; however, immunocytochemistry allowed us to correlate the relative levels of nuclear Hsf1 with β-catenin and Hsp90 using serial tissue sections. Dysplastic regions were identified by elevated levels of β-catenin (Figure 7A). Using adjacent sections, we observed clear nuclear enrichment of Hsf1 in cells associated with high levels of both β-catenin and Hsp90 (Figure 7B–7C and inset 1). Importantly, in normal cells adjacent to the dysplasia with high levels of Hsp90, but normal levels of β-catenin, we also found clear evidence of nuclear Hsf1 (Figure 7A–7C, inset 2). In cells with lower levels of Hsp90, we found generally lower levels of nuclear Hsf1 (Figure 7A–7C, inset 3). These patterns support our *in vitro* findings that Hsf1 drives the increases in HSPs observed in normal cells carrying a single mutation in APC and that these changes are preserved during cancer progression.

## DISCUSSION

We have identified a novel link between a cancer driver mutation in the tumor suppressor gene, *adenomatous polyposis coli*, and the activation of a cell stress pathway, a state that precedes cancer in the intestine but also anticipates the mature cancer phenotype found in carcinomas. In APC^Min/+^ intestines we find that the a cell stress pathway is activated in histologically normal crypt cells and is further activated in cells adjacent to or within regions that have experienced loss of full length APC. We establish that chronic inhibition of microtubule dynamics represents the underlying mechanism that links APC mutants to an HSF1-dependent cell stress response that is distinct from the canonical heat shock response. The action of a monoallelic mutation of APC to induce global changes in otherwise normal cells raises the possibility that HSF1-mediated changes act early in cancer onset.

Cancer onset is largely attributed to the presence of so-called driver mutations identified in studies of mature tumors that can, when introduced into normal cells, induce the changes required for cancer initiation [39, 52–55]. However, the molecular details that accompany the transition of normal cells to cancer cells remain unclear. One idea is that early changes in normal cells lead to a “permissive” environment that precedes cancer cell transformation. The “cancer field effect”, for example, posits that there are molecular genetic changes found in histologically normal cells that border and precede cancer cell transformation that contribute to oncogenesis [40–42]. Our results implicate Hsp90 as a novel marker of the cancer field effect. Hsp90 levels are clearly elevated in cells that border the small dysplastic lesions defined by their loss of APC and increase in β-catenin (see Figures 1 and 2). Changes in these border cells might be induced by neighboring cancer cells or could by cell autonomous changes, potentially brought on through increased genome instability [43, 44]. We favor the idea that the Hsp90 increase is independent of nearby cancer cells, as Hsp90 levels are already elevated in normal crypt cells quite far from the nearest dysplastic lesion (see Figure 3). It is possible that increases in Hsp90 are linked to the chromosome instability previously shown to occur in cells expressing the APC^Min^ mutation [28]. However, the incidence of mitotic errors is much less frequent than the incidence of cells with elevated Hsp90. In addition, our *in vitro* work suggests that Hsp90 levels increase due to cytoskeletal perturbations and not to genomic damage. We favor the idea that Hsp90 may be a common marker of cells in the cancer field and that the activation of the cell stress pathways precedes genomic instability, possibly providing a “buffer” to mutations that arise later or to proteomic stress caused by aneuploidy as suggested by Lindquist and colleauges [45–48].

The activation of cell stress pathways is an evolutionarily conserved response to changes in cellular homeostasis typically studied after exogenous insults, such as heat shock. There is also evidence that HSPs provide important functions for cells in response to intrinsic signal transduction pathways. For example, the p38-stress kinase is activated downstream of growth factors and targets Hsp27 to help stabilize actin filaments [49]. Studies of endothelial barrier function have implicated Hsp90 in regulating the Hsp27 response following activation of signaling pathways or drug treatments that induce microtubule depolymerization [50, 51]. These studies and others have provided evidence for cross-talk between microtubules and the actin cytoskeleton, perhaps most relevant is the interaction between EB1, APC and Rho to regulate actin polymerization through formins [52–55]. Although the details underlying these interactions are far from clear, it is apparent that microtubule dynamics impact actin organization that in turn triggers Hsp27 in a regulatory circuit that likely includes the Hsp90 and Hsf1. Thus, perturbation of microtubule dynamics by the clinically relevant APC truncated proteins may trigger a large network of signals that alter microtubules, actin and ultimately nuclear activities that change the transcriptional landscape. We speculate that chronic activation of such pathways provides a survival advantage in evolving pre-cancer cells as secondary genomic alterations accumulate (e.g., loss of hetrozygostiy at APC). Additional characterization of the pathways affected by APC mutants and the downstream changes will be critical in assessing the validity of such a model.

Hsf1 is the master regulator of the heat shock response and our findings argue that APC mutants associated with human colorectal cancer activate Hsf1. We find an increase in mRNA from HSP genes that regulated by Hsf1 and chemical inhibition of Hsf1 prevents the accumulation of HSPs after inhibition of microtubule dynamics *in vitro*. Remarkably, any of the conditions used to inhibit microtubule dynamics also causes an enrichment of Hsf1 in the nucleus of RPE-hTERT cells. Although the nuclear enrichment is required for its transcriptional activity, the regulatory scheme for Hsf1 nuclear import and transcription indicates that an inactive pool of Hsf1 can be imported into the nucleus, arguing that nuclear import and transcription are separable [2, 56]. This may explain why siRNA against EB1 causes Hsf1 to become enriched in the nucleus without a corresponding increase in HSP mRNA. One potential outcome of Hsf1 activation is that cells with chronic perturbations in microtubule dynamics will have increased levels of HSPs; this may provide for a more robust environment that can support malignant transformation events. In this case, increases in Hsp90 may serve to stabilize active Ras mutants or may prevent p53 activation, steps that presumably must occur at some frequency for intestinal cancers to develop [1, 57]. Alternatively, Hsf1 may have more direct role in deregulating cell growth and division pathways that contribute to inappropriate cell division. Mendillo and colleagues showed that in cancer cells, Hsf1 targets promoters that are distinct from those it binds to after heat shock. A dramatic case in point is the gene encoding for Hsp70. In cancer cells, Hsp70 mRNA and Hsf1 binding at the promoter is low compared to other regions, suggesting a distinct Hsf1-mediated transcriptional program [2, 5–8]. We also find that Hsp70 does not respond to mutations in APC or inhibition of microtubule dynamics, suggesting this response is more akin to the Hsf1-mediated cancer program than a canonical heat shock program. Thus, a single APC mutant may induce an Hsf1 program that anticipates cancer.

In summary, this work sheds light on a novel pathway that links APC mutants found in cancers to early activation of a cell stress pathway, that target a network of genes and proteins that have a well-established role in modulating cell fate. The important implication is that early activation of this cell stress network may aid the transition of a normal cell into a cancer cell, one that continues to evolve malignant phenotypes. Significantly, this work also provides a novel set of biomarkers to expand our definition of potentially cancer prone tissue. Our findings help establish a novel path to explore the changes that precede cancer cell transformation and how cell stress pathways contribute to cancer initiation and progression.

## MATERIALS AND METHODS

### Cell culture, transfection, siRNA, and inhibitor treatments

Human hTERT-RPE cell lines, obtained from ATCC, were grown in high glucose Dulbecco's Modified Eagle's Medium supplemented with 5% fetal bovine serum, sodium pyruvate, GlutaMAX, and Penicillin/Streptomycin (Life Technologies, Carlsbad, CA), with media changed every 2–3 days.

For transfection experiments, a CMV-driven 13-myc-APC^1–1450^ plasmid was constructed as previously described (Caldwell and Kaplan, 2007). RPE cells were plated to approximately 80% confluence in 6-well culture dishes and allowed to grow overnight under standard growth conditions. Cells were then transfected with 13-myc-APC^1–1450^, or control plasmid, using Mirus (Mirus Bio, Madison, WI) transfection reagent according to the manufacturers instructions. Cells were harvested for immunoblot or immunofluorescence 48–72 hours post-transfection.

For siRNA experiments, cells were split into 6-well culture dishes, then grown overnight under standard conditions in OptiMEM media (Life Technologies, Carlsbad, CA). The next day a custom EB1 siRNA, sequence GUGAAAUUCCAAGCUAA GCdTdT (GE Heathcare Dharmacon, Lafayette, CO), was prepared to the indicated final concentration per well in Oligofectamine (Life Technologies, Carlsbad, CA) according to the manufacturers instructions. The cultures were grown overnight, FBS was then added to a final concentration of 5%, and the cultures were then allowed to grow an additional 48 hours under standard conditions before harvesting for immunofluorescence or western blotting.

For nocodazole experiments, hTERT-RPE cells were plated to approximately 60% confluency in 6-well culture dishes and allowed to grow overnight under standard growth conditions. Nocodazole (Sigma-Aldrich, St Louis, MO), or DMSO vehicle control, was added at the indicated concentration and cells were harvested 12 hours post-treatment. For Hsf1 inhibition experiments, KRIBB11 (EMD Millipore, Billerica, MA), or a DMSO vehicle control, were added alone at indicated concentrations, or simultaneously with nocodazole, and harvested 12 hours post-treatment.

### Western blotting

Cell extracts were prepared using a triton X-100 lysis buffer and the soluble fraction was isolated as previously described [10–12, 58]. A Bio-Rad Protein Assay (Bio-Rad, Hercules, CA) was used to determine the protein concentration and a total of 40ug soluble extract was added to each lane of a 4–12% gradient Bis-Tris polyacylamide gel and run in MES buffer (Life Technologies, Carlsbad, CA). The gel was then transferred overnight to a 0.1 uM nitrocellulose membrane and processed as previously described [14, 58]. Primary rabbit anti-Hsp90 alpha, beta, or pan at 1:500 as indicated (Enzo Life Sciences, Farmingdale, NY), rabbit-anti-Hsp70 1:1000, mouse anti-Hsp27 1:1000, or rabbit anti-Hsp27 pS85 1:1000 (Cell Signaling Technology, Danvers, MA), mouse anti-tubulin 1:2000 (Abcam, Cambridge, MA), and secondary goat anti-rabbit IRDye 680CW and goat anti mouse IRDye 800CW 1:10, 000 (Licor, Lincoln, NE) were use according the manufactures directions. Images were collected using a Licor Odyssey system. Signal intensities were measured using ImageJ software (http://imagej.nih.gov/ij/). and analyzed using statistical analysis software as outlined below.

### Mouse tissues

Wild-type and Min mouse tissues were obtained in accordance with approved UC Davis Institutional Animal Care and Use Committee protocols as previously described [18, 28]. Mouse small intestinal tissues were harvested from 20-week old mice and genotyped. Intestinal tissues were then fixed in 2% formaldehyde and paraffin embedded. A total of 9 genotyped mice were used for each experiment (4 wild type and 5 Min heterozygotes).

### Immunofluoresence of cell culture and tissue samples

Cell lines were plated on ethanol rinsed cover slips, then cultured as described above. The cells were grown overnight, media removed, and rinsed in phosphate buffered saline (PBS) pH 7.2 then fixed for 20 minutes in 3.7% formaldehyde in PBS at 37°C as previously described [6, 21, 23, 58].

Five micron tissue sections were cut from paraffin embedded mouse intestine. The cut sections were then deparaffinized using three xylene washes and three ethanol washes (100%, 95%, 70% ethanol, respectively), then rinsed in tap water for five minutes. Antigen retrieval was conducted in 10mM Citrate Buffer with 0.1% tween for 30 minutes at 95C. Tissues were then treated with hydrogen peroxide to quench endogenous peroxidase treatment, if 3, 3′ diaminobenzadine staining was to be conducted, or sections were blocked and stained for immunofluorescence as described above. For 3, 3′ diaminobenzidine staining the VECTASTAIN Elite ABC kit was used according to manufacturers directions (Vector Laboratories Inc., Burlingame, CA).

For cell culture and mouse tissue staining experiments, rabbit anti-pan Hsp90 was used at 1:100 (Enzo Life Sciences, Farmingdale, NY), mouse anti-β-catenin at 1:200 (BD Biosciences, San Jose, CA), rabbit anti-cAPC at 1:100 (Produced in-house as previously described (Caldwell and Kaplan, 2007) diluted in blocking buffer. For cell culture staining experiments mouse anti-Hsp27 was used at 1:200, rabbit anti-HSF1 at 1:200 (Cell Signaling Technology, Danvers, MA), or mouse anti-EB1 at 1:100 (BD Biosciences, San Jose, CA) were diluted in blocking buffer. Secondary Alexa Fluor 488 goat anti-rabbit or Alexa Fluor goat anti-mouse antibodies (Life Technologies, Carlsbad, CA) were diluted 1:100 in blocking buffer. Both cell culture and tissue section samples were stained with DAPI 1:10,000 then mounted in glycerol containing p-phenylenediamine then sealed with clear nail polish.

### Image collection/analysis and statistical analysis

Cell culture and tissue section sample images were collected using either a Nikon E600 epifluorescence microscope fitted with a 60X (NA = 1.4) oil immersion lens (Nikon Instruments, Melville, NY), and collected on a Hamamatsu Orca ER charge-coupled device camera (Hamamatsu, Bridgewater, NJ) with ImageJ software, or on an Applied Precision Deltavision Deconvolution microscope fitted with a 60X (NA = 1.4) or 10X (NA = 0.4) oil immersion lens (Applied Precision GE Healthcare, Issaquah, WA) and collected with a Nikon Cool-Snap CCD camera (Nikon Instruments, Melville, NY). Images were deconvolved using Applied Precision software and then transferred to ImageJ for further analysis. Image signal intensity measurements were performed in ImageJ as previously described (Davies and Kaplan, 2010) using the average “integrated intensity” within a fixed region size across all images analyzed. Comparisons of integrated intensities were made using a region of interest of fixed area across each sample. Integrated intensity measurements were then plotted using Microsoft Excel and analyzed for statistical significance using a 2-tailed *T*-test; values < 0.05 were considered statistically significant. All experiments were replicated at least 3 times.

### Real-time quantitative polymerase chain reaction

Cell culture and treatments were performed as described above. Cell lysis and RNA extraction was performed using the Qiagen RNeasy Plus Mini Kit (Qiagen, Germantown, MD) according to the manufacturers instructions. Total RNA concentration was measured spectrophotometrically and equal concentrations were added to each well of a 96-well PCR plate. RT-qPCR was performed using the Bio-Rad iTaq reagent in a Bio-Rad CFX96 (Bio-Rad, Hercules, CA). Hsp90 probe sequence (5′ to 3′) TTCAGACAGAGCCAAGGTGC, Hsp27 probe sequence GGCATTTCTGGATGTGAGCC, and GAPDH probe sequence GGGAGCCAAAAGGGTCATCATCTC. Data analysis was performed with the manufacturer-supplied software.

## SUPPLEMENTARY FIGURES


