# Supplementary material for: Adenomatous polyposis coli mutants dominantly activate Hsf1-dependent cell stress pathways through inhibition of microtubule dynamics
Source: Oncotarget. 2015 Jul 22;6(28):25202–16. doi: 10.18632/oncotarget.4513 (PMC4694825; doi:10.18632/oncotarget.4513)
Supplement: Supplementary file 1 [file oncotarget-06-25202-s001.pdf]

SUPPLEMENTARY FIGURES

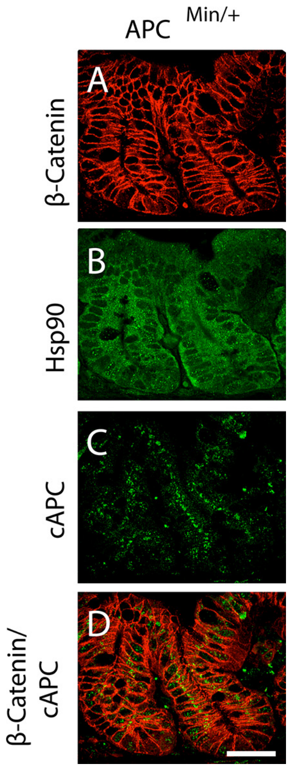

Supplementary Figure 1: Normal crypt cells in APC$^{Min/+}$ with elevated Hsp90 exhibit wild type levels of β-catenin and expression of full length APC. A. C. and **D.** APC$^{Min/+}$ small intestinal tissue section that is histologically normal, and > 10 crypts from an intestinal dysplasia was stained with antibodies against β-catenin and full length APC. **B.** A serial section from the same region was also stained with antibodies to Hsp90. Despite an approximately 1.5-fold elevation in Hsp90 (see Figure 3), this region exhibited normal levels of (A) β-catenin and expression of full length APC (C; cAPC). (D) The images from cAPC (green) and β-catenin (red) co-staining were merged. Scale bar = 20 uM.

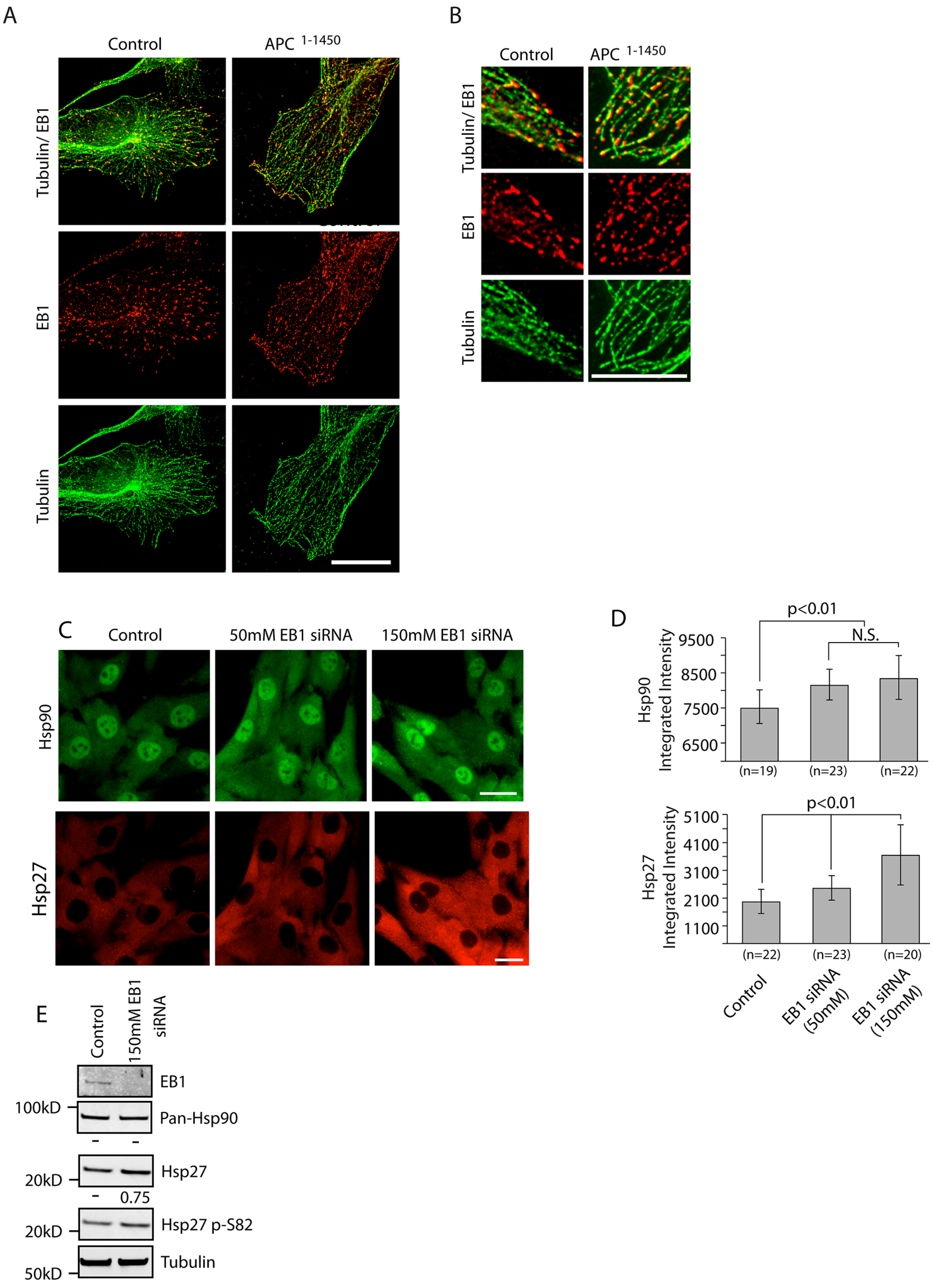

Supplementary Figure 2: EB1 changes in cells expressing APC$^{1-1450}$ and EB1 inhibition activates cell stress pathways. **A.** hTERT-RPE cells were transiently transfected with CMV-myc (Control) or CMV-myc-APC$^{1-1450}$ (APC$^{1-1450}$) and stained with antibodies against tubulin and EB1. Scale bar = 20 uM. **B.** Magnified views of cortical regions from the images displayed in (A) illustrate microtubule organization and EB1 association with plus-ends and microtubule walls. **C.** hTERT-RPE cells treated with the indicated siRNA were fixed and stained 72 hours later for Hsp90 and Hsp27 as indicated. **D.** The fluorescence intensities for Hsp90 and Hsp27 was quantified as previously described and statistically significant differences are annotated with the *p*-value or N.S. if the there was no significant difference. Scale bar = 20 uM. **E.** Immunoblots were performed and signals quantified on hTERT-RPE cell extracts following the indicated siRNA treatments. Fold-change relative to controls are provided below relevant samples. No change is indicated by a "-".

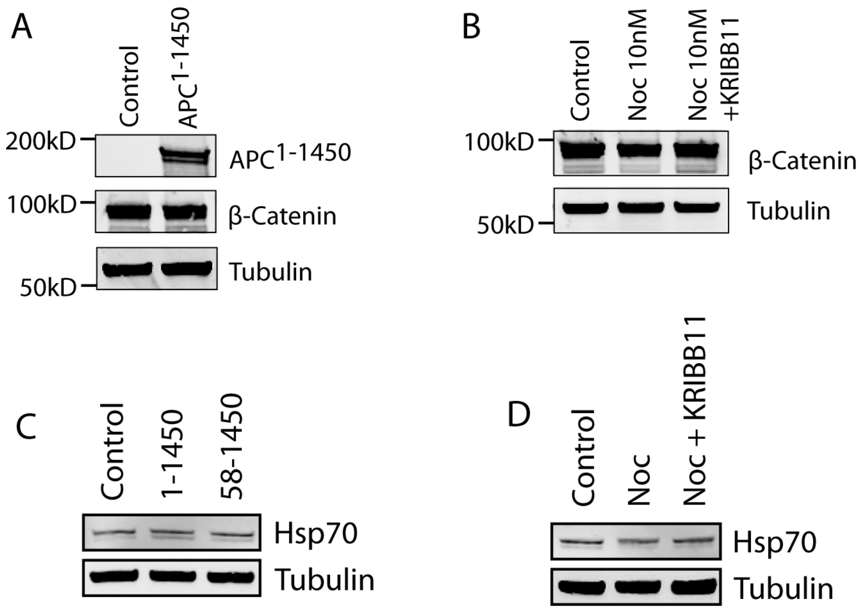

Supplementary Figure 3: Transient *in vitro* expression of APC$^{1-1450}$ or treatment with nocodazole do not affect β-catenin levels. A. Immunoblot of hTERT-RPE cell extracts showing APC$^{1-1450}$ and β-catenin expression levels, compared to tubulin as a loading control, following transient transfection with CMV-myc (Control) or CMV-myc-APC$^{1-1450}$ (APC$^{1-1450}$) containing plasmids. **B.** Immunoblot of hTERT-RPE cell extracts for β-catenin following treatment with vehicle or the indicated concentrations of nocodazole and KRIBB11. Molecular weight marker migration is indicated on the left portion of the images. **C.** and **D.** Immunoblots of Hsp70 levels following transfection with APC mutants, treatment with nocodazole, or nocodazole and the Hsf1 inhibitor KRIBB11.

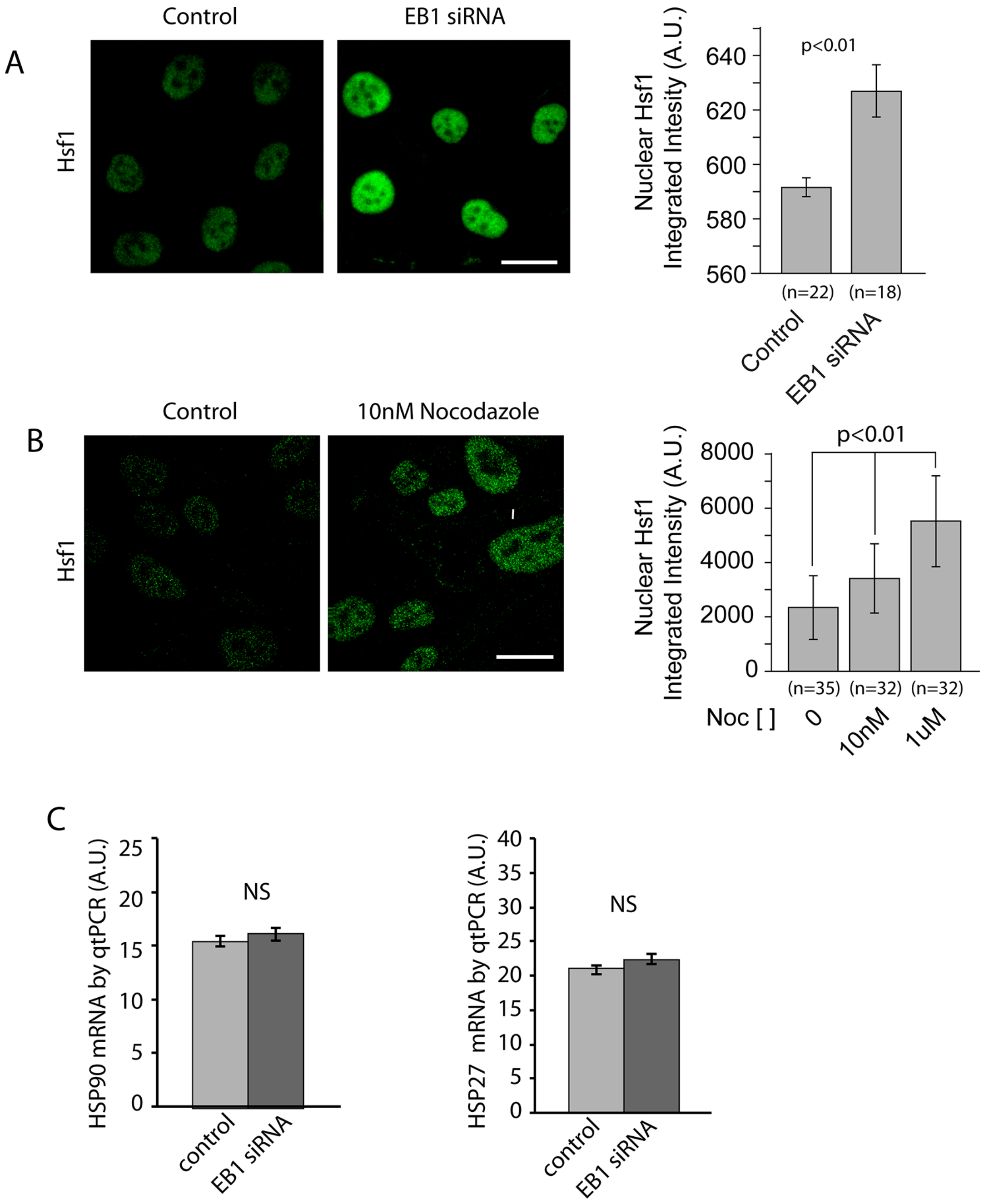

Supplementary Figure 4: Hsf1 is enriched in hTERT-RPE cell nuclei following siRNA inhibition of EB1 or treatment with low dose nocodazole. hTERT-RPE cells were stained with Hsf1 antibodies after transfection with **A.** control or EB1 siRNA as indicated or with DMSO (carrier control) or **B.** 10 nM nocodazole, as indicated. Total integrated fluorescent intensities were measured for nuclear Hsf1 for both conditions as described in other figures and in Materials and Methods. The number (n) of cells measured are indicated below each treatment. Scale bar = 20 uM. **C.** Hsp90 and (F) Hsp27 mRNA levels were measured using RT-qPCR after transfection with control siRNA or with EB1 specific siRNA, as indicated. Levels of GAPDH mRNA were used to standardize the total amount of mRNA between samples and levels indicate relative levels of the indicated mRNA.
